# The Interplay between Natural Killer Cells and Human Herpesvirus-6

**DOI:** 10.3390/v9120367

**Published:** 2017-12-01

**Authors:** Eva Eliassen, Dario Di Luca, Roberta Rizzo, Isabel Barao

**Affiliations:** 1 HHV-6 Foundation, Santa Barbara, CA 93108, USA; eva@hhv-6foundation.org; 2 Section of Microbiology, Department of Medical Sciences, University of Ferrara, 44121 Ferrara, Italy; ddl@unife.it; 3 Department of Microbiology and Immunology, University of Nevada, Reno, NV 89557, USA

**Keywords:** NK cells, HHV-6, disease, immune cells, infection

## Abstract

Human Herpesvirus 6 (HHV-6) is a set of two closely related herpes viruses known as HHV-6A and HHV-6B. Both are lymphotropic viruses that establish latency in the host. The ability to evade the immune responses of effector cells is likely a major factor contributing to the development of a persistent HHV-6A/B (collectively termed HHV-6) infection. Natural killer (NK) cells are lymphocytes that, along with neutrophils and monocytes/macrophages, participate in the critical innate immune response during viral infections, but can also mediate the antigen-specific memory responses generally associated with adaptive immunity. NK cells compose the first barrier that viruses must break through to continue replication and dissemination, and a weak NK cell response may predispose an individual to chronic viral infections. Both HHV-6A and HHV-6B can interfere with NK cell-mediated anti-viral responses but the mechanisms by which each of these viruses affect NK cell activity differs. In this review, we will explore the nuanced relationships between the two viruses and NK cells, discussing, in addition, relevant disease associations.

## 1. Introduction

Human herpesvirus 6 (HHV-6) is a member of the *Herpesvirales* order, *Herpesviridae* family, *Betaherpesvirinae* subfamily, and *Roseolovirus* genus. HHV-6 is classified into HHV-6A and HHV-6B as two distinct species [1]. The term HHV-6 remains in usage and collectively refers to the two species.

HHV-6 exhibits wide cell tropism in vivo and, as with other herpesviruses, induces a lifelong latent infection in humans (Table 1). HHV-6 preferentially replicates in activated CD4+ T lymphocytes [2,3] and uses specific cell receptors permitting virus anchorage to the cell surface: HHV-6A uses CD46, a regulator of complement activation expressed on all nucleated cells, while CD134 (also called OX40), a member of the tumor necrosis factor (TNF) receptor superfamily present only on activated T lymphocytes, functions as a specific entry receptor for HHV-6B [4,5]. In addition to CD4+ T lymphocytes, HHV-6 can infect in vitro CD8+ T lymphocytes (only with HHV-6A), human fibroblasts, natural killer (NK) cells, liver cells, epithelial cells, endothelial cells, astrocytes, oligodendrocytes, and microglial cells [2,6,7,8,9,10,11,12,13]. The host tissue range of HHV-6 in vivo appears to be broader than might be expected from in vitro studies and includes the brain, tonsils, salivary glands, kidneys, liver, lymph nodes, heart, lungs, gastrointestinal tract, and monocytes/macrophages [2,14,15,16]. The preferential sites for virus latency are suspected to be monocytes/macrphages, bone marrow progenitors and central nervous system (CNS) cells [17,18,19].

As a key difference with other human herpesviruses, HHV-6 DNA can be integrated into the subtelomeric region of chromosomes in every nucleated cell of the body, including germ cells. This condition, known as inherited chromosomally integrated HHV-6 (iciHHV-6/ciHHV-6), is present in about 1% of the general population and is vertically transmitted [20]. 

## 2. HHV-6 Epidemiology and Infection

HHV-6A and HHV-6B are ubiquitous viruses [2] that are detected in more than 90% of adult populations in developed countries. Primary infection usually occurs very early in life, between 6 months and 2 years of age, following the loss of protective maternal antibodies [32]. Generally, primary HHV-6B infection is accompanied by fever and a rash (exanthema subitum). At an even earlier stage of life, congenital infection following intrauterine transmission or germ line transmission of ciHHV-6 (i.e., congenital ciHHV-6) can occur and has been reported in about 1% of children. It is generally believed that in the majority of countries, primary HHV-6B infection occurs first, and in many cases is associated with clinical symptoms, whereas HHV-6A is acquired later, through asymptomatic infection. 

Saliva is assumed to be the main vehicle for virus transmission, as supported by the frequent detection of HHV-6 in saliva and salivary glands. Virus transmission through organ transplantation has been described infrequently, while blood transfusion and breastfeeding have never been reported as the origins of primary infections [33,34].

The clinical symptoms associated with HHV-6 reactivations, which are most commonly reported in transplant recipients, include fever, rash, and transiently decreased numbers of circulating blood cells belonging to the granulocyte/macrophage, erythroid, and megakaryocytic lineages [35,36,37]. In addition, subacute limbic encephalitis and delayed engraftment are now recognized as typical opportunistic diseases due to HHV-6 reactivation in hematopoietic stem cell transplant (HSCT) recipients [31]. While the effects of chronic HHV-6 infection are yet to be fully understood, the virus has been implicated as a possible trigger for multiple sclerosis (MS) [38,39], chronic fatigue syndrome [40,41,42], myocarditis and subsequent chronic cardiomyopathy [30,43,44,45], Hashimoto’s thyroiditis (HT) [46], and, through interference with the normal status of endometrial NK cells, primary idiopathic female infertility [21]. 

## 3. HHV-6 Proteins

Roseolovirus genomes are approximately 65 to 90 kb shorter than the 235 kb human cytomegalovirus (HCMV) genome. HHV-6A and HHV-6B are ~90% identical across their genomes, with ~95% of their identity conserved across the herpesvirus core genes. When the viral genome is released into the nucleoplasm, the virus uses the cellular transcription and translation machinery to produce three kinetic classes of viral proteins (immediate early (IE), early (E), and late (L) (Table 2). 

HHV-6 DNA replication requires seven virally encoded factors. First, the origin binding protein, encoded by the *U73* gene, binds to the origin of lytic replication (ori-lyt) and denatures a portion of the circular viral DNA genome [47]. This gap is maintained by the helicase/primase complex, consisting of the *U43*, *U74* and *U77* gene products, which also provides RNA primers for the lagging-strand DNA synthesis [48]. The single-stranded DNA in the “replication bubble” is stabilized by the major DNA binding protein, encoded by *U41*, until second-strand synthesis is catalyzed by the DNA polymerase, pU38 [49], driven by a processivity factor, pU27 [50]. The four proteins encoded by the *U79* and *U80* genes of HHV-6 are suspected of being involved in DNA replication as well, although their roles are not yet understood [51]. As the new strand grows, the circular replication structure is nicked to form a rolling circle intermediate. Long concatameric strands of progeny DNA are encapsidated by the interaction of cleavage and packaging proteins with specific packaging (pac) signals at the end of the viral genomes. Notably, ori-lyt and pac sequences are different for HHV-6A and HHV-6B [52]. The mature capsids bud out of the nucleus (thereby temporarily acquiring an intermediate membrane devoid of glycoproteins) into the cytoplasm, where they acquire a tegument and a secondary spiked viral envelope at the Golgi complex or at annulate lamellae, where viral glycoproteins accumulate. These are sequentially glycosylated in transport vesicles prior to the release of mature virus particles into the extracellular space by exocytosis. The HHV-6 maturation pathway is different from that of the other herpesviruses in that no viral glycoproteins are detectable in the cell membrane of infected cells [53]. The total time from infection to release of new virions takes approximately 72 h.

Like the other human herpesviruses, HHV-6 is capable of persisting in the host after primary infection, and non-productive infection is characterized by the presence of latency-associated transcripts [54]. The *U94* gene product, the expression of which has impaired the migration of endothelial cells [5] and human oligodendrocyte progenitor cells in vitro [55], may enable the establishment and/or maintenance of latent infection [56]. Although HHV-6 has primarily been studied in cases of acute productive infection, as in instances of drug-induced hypersensitivity syndrome/drug reaction with eosinophilia and systemic symptoms (DIHS/DRESS) and reactivation in solid organ and HSCT recipients, the ability of the virus to induce changes in its environment in the absence of strong episodes of replication [57,58] has spurred growing interest in chronic, semi-latent HHV-6 infections. As HHV-6 is able to infect a great range of cells in vivo, pathogenic effects of chronic infections could have far-reaching consequences.

## 4. Interactions with the Immune System

**Figure 1:**
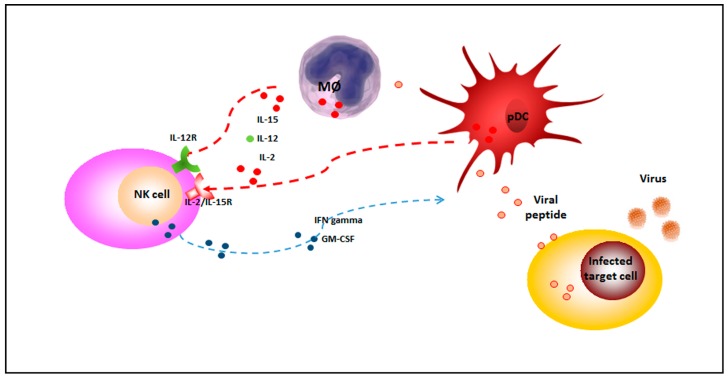
NK cells during viral infections. During viral infections, NK cells are activated by IL-2, IL-12 and IL-15, released by plasmacytoid dendritic cells (pDC) and monocytes/macrophages (MØ) (red dotted lines) and secrete potent cytokines, such as interferon gamma (IFN-gamma), that lyse susceptible targets and enhance innate and adaptative immune responses (blue dotted line). NK: natural killer; IL-2/IL-15R: IL-2/IL-15 receptor; IL-12R: IL-12 receptor; IFN-gamma: Interferon gamma; GM-CSF: Granulocyte-macrophage colony-stimulating factor.

HHV-6 is able to both stimulate and modify immune responses [2]. Prior to the emergence of adaptive immunity, HHV-6 stimulates the effectors of innate immunity; an increased secretion of proinflammatory cytokines, such as interleukin-1 beta (IL-1beta), TNF-alpha, and alpha interferon (IFN-alpha), is observed in peripheral blood mononuclear cells, and NK cell activity associated with IL-15 synthesis increases during HHV-6A infection [59,60]. Moreover, HHV-6 has been shown to promote a cytokine profile shift from Th1 to Th2 by upregulating IL-10 and human leukocyte antigen-G (HLA-G) expression [61] while downregulating HLA class I [6] and IL-12 [62,63]. Blocking IL-12 abolishes dendritic cell (DC)-induced IFN-gamma secretion by NK cells (Figure 1) [64]. On the other hand, IL-15 upregulation by DC and monocytes/macrophages stimulates NK cell IFN-gamma secretion. These effects are mediated by HHV-6-encoded proteins which act as analogues of cell chemokines and are believed to promote viral growth, viral dissemination, and/or escape from the immune response. Specifically, HHV-6 encodes putative chemokines and chemokine receptors and induces the production of cytokines that are able to modify the immune response. 

## 5. NK Cells and HHV-6 Infection

### 5.1. NK Cells

Evidence indicates that HHV-6 alters NK cell activity by affecting their ability to control viral infection. As NK cells are known to represent an important natural defense mechanism in controlling viral infection, evasion of the NK cell response could confer considerable benefits to the virus [65,66,67].

NK cells, like B and T cells, are a lymphocyte lineage derived from the common lymphoid progenitor (CLP), and like B cells, they are thought to develop primarily in the bone marrow. NK cells also undergo an “education” process during development, during which they acquire the ability to recognize lack of self HLA class I, or “missing-self”, a feature that facilitates their surveillance of target cells that have downregulated HLA class I during infection or malignancy. NK cells rely on both cytokines and transcription factors to promote and control their development. Cytokine signaling from IL-15 is critical for the development of NK cells and is required throughout their lifetime. 

Plentiful studies in the mouse and human demonstrate NK-cell-dependent protective effects during infections with coxsackievirus, human immunodeficiency virus (HIV), hepatitis C virus (HCV), influenza virus, and poxvirus, and herpesviruses [68,69,70]. Their direct antiviral effects include killing of infected target cells and production of interferon γ (IFN-γ) [70]. The antiviral responses are regulated by a repertoire of germline encoded NK cell receptors (NKRs) recognizing ligands on virus-infected cells and by innate cytokine responses induced during infections.

Inhibitory receptors signal via immunoreceptor tyrosine-based inhibition motifs (ITIMs) located in their cytoplasmic tails. These types of signals predominate under normal conditions, as the majority of target cells express HLA-I molecules. When target cells are transformed or infected, surface expression of HLA-I molecules can be abrogated; the absence of inhibitory NKR ligation allows the NK cell to kill its target (via “missing-self” recognition). In addition to a requirement for release from dominant inhibitory signaling, effective NK cell killing also requires the ligation of activating NKRs. Activating NKRs lack ITIMs, but they associate with membrane-bound adaptor molecules, such as DAP12 (12 kDa transmembrane transduction receptor element) (i.e., Ly49, KIR (killer cell Immunoglobulin-like receptors)), FcεRγ, and CD3ζ (CD16, NKp46), that bear immunoreceptor tyrosine-based activation motifs (ITAMs) [71]. Interestingly, a KIR2DL4 receptor can have both inhibitory and stimulating activity. 

### 5.2. NK Cells Role in HHV Infections

Herpesviruses employ mechanisms used to evade host-specific immunity. HCMV, for instance, downregulates HLA class I expression by using several different proteins in an effort to evade CMV-specific CD8+ T cell responses. HCMV also encodes for several HLA class I homologs that engage inhibitory KIRs to avoid NK cell activation. Additional HCMV proteins serve as ligands for other inhibitory NK cell receptors. One such protein is UL18, which binds to the inhibitory leukocyte immunoglobulin-like receptor 1 (LIR-1) expressed on NK cells, thereby dampening LIR-1+ NK cell function [71,72]. Furthermore, HCMV expresses several proteins that downregulate the expression of activating NKRs and their ligands [73,74,75,76].

### 5.3. NK Cells in HHV-6 Infections

Taking into consideration the fact that other herpesviruses (i.e., HCMV and Epstein–Barr virus) are able to remodel NK cell compartments [77,78], the relationship between HHV-6 and NK cells has emerged as an intriguing field of study. At this time, there are few studies on the consequences of HHV-6 infection on NK cell activity per se, in large part due to the absence of suitable animal models. However, the literature as it stands indicates that the two are strongly interconnected. Upon infection, NK cells act to control the dissemination of the virus, lysing autologous HHV-6-infected PBMC (peripheral blood mononuclear cells) [79]. Accordingly, during the acute febrile phase of HHV-6 infection, there is a significant increase in IFN-α and NK activity when compared to the convalescent phase [59,80]. The heightened NK cell activity is enhanced by the interactions between both IL-15 and CD122 and the beta subunit of the IL-2 receptor. It is known that IL-15 is able to increase the cytotoxicity of NK cells [81], and, in turn, induce the production of the anti-viral factor IFN-γ by CD4+ and NK cells [82]. 

HHV-6 can also infect NK cells [83]. It has been reported that NK cells rapidly internalize HHV-6 (i.e., within 1 h) but signs of productive infection are only observed if cells are cultured for several weeks prior to infection, a time at which no residual NK cytotoxic activity is observed [83]. Interestingly, infection of NK cells by HHV-6 also leads to de novo expression of CD4, an antigen not expressed by NK cells, thereby predisposing these cells to infection by HIV-1. These results provide evidence that direct infection of NK cells may represent a potential strategy to suppress the natural anti-viral immunity of the host. 

In vivo studies revealed an increase in the cytotoxicity and percentages of the CD56brightCD16neg/dim NK cell subset in patients with Hashimoto’s thyroiditis (HT), a disease in which HHV-6A has been considered as a potential contributor [29,46,84]. These results suggest that the NK cells of patients with HT might be inherently altered to respond to HHV-6A infection with high levels of cytotoxicity, thereby encouraging the development of autoimmunity and furthering the disease. Moreover, the myriad cytokines produced by activated NK cells and direct intercellular interactions can activate dendritic cells, NKT (Natural killer T) cells, B cells, and CD4+ T cells [85], as well as autoreactive T cells [85] that can lead to autoimmune reactions. Indeed, elevated NK cell cytotoxicity has been found in a range of autoimmune disorders, including rheumatoid arthritis [86], autoimmune diabetes [87], and primary biliary cirrhosis [88], where a possible role of viral infection in pathological exacerbation is suggested [89,90,91]. 

**Figure 2:**
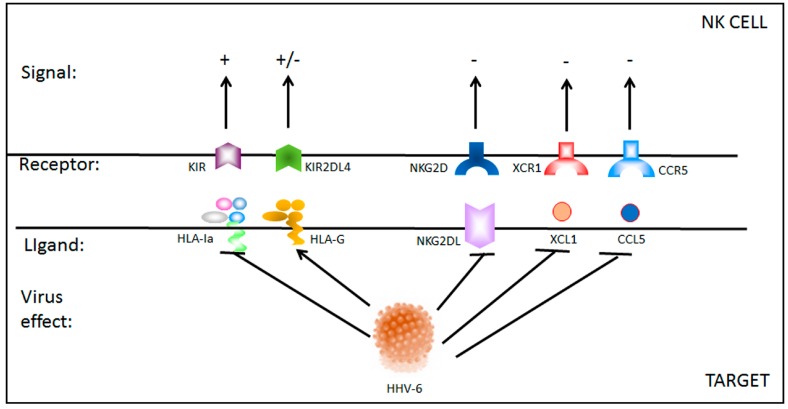
HHV-6 effect on NK cell receptor–ligand interaction. During HHV-6 infection, both NK cells and infected target cells present a modified pattern of receptor–ligand expression, that affect NK cell status. KIR: killer immunoglobulin-like receptors with inhibitory functions; KIR2DL4 with both inhibitory and activating functions; NKG2D activating receptor; XCR1 and CCR5 (C-C chemokine receptor) chemokine receptors. Virus effect: arrow means activated, T bar means inhibited; signal: + means activating signal, +/− means both activating and inhibitory signal, − means inhibitory signal.

On the other hand, CD56bright NK cells have been found to be helpful in combatting symptoms of autoimmunity, as in some patients with multiple sclerosis (MS) in whom NK cells reduce demyelination through the suppression of T cells [92,93]. This presents a conflicting view of NK cells, in which they are beneficial at times in mitigating an infection or a disease, especially at the onset of an infection, yet at other times, they exacerbate the pathological response, presumably through dysfunctional activity. Both the time point and the functions of the NK cell subset are therefore of key importance in elucidating the course of infection and the development of the disease. Tissue-resident NK cells, for example, possess unique features, such as different receptor repertoires, in comparison to their peripheral blood counterparts, and this variation in cellular characteristics leads to differing downstream effects. Consequently, HHV-6 infection may affect tissue-resident CD56bright NK cells while peripheral blood CD56bright NK cells are not affected in the same manner. In a prime example of this phenomenon, results from a recent study demonstrated a reduction in the CD56brightCD16neg/dim NK cell subset resident in the endometrium of idiopathic infertile women with a concurrent endometrial HHV-6A infection [21]. The uterine NK cells from 43% of women infected with HHV-6A also displayed ex vivo increased activation and degranulation (CD107a expression) towards HHV-6A infected cells, indicating that specific receptor-antigen recognition and signaling (e.g., KIRs-HLA and NKG2D-MICs (MHC class I polypeptide-related sequence), etc.) may be modified by the presence of HHV-6 infection (Figure 2). In contrast, peripheral blood NK cell levels were not significantly different among women with HHV-6A and those without the virus.

## 6. HHV-6 Control of NK Cell Responses

HHV-6 early antigens are involved in regulating NK cell responses (Table 3). The expression of the U51A HHV-6A viral receptor in concert with the expression of the HHV-6A ligand U83A affects the binding of ligands to NK activating receptors. U51A binds the ligand XCL1 (Chemokine (C motif) ligand), which normally binds to the CCR7 receptor on CD56brightCD16neg NK cells and the XCR1 receptor on the CD56dimCD16pos NK cells [94]. This may lower the ability of receptor–ligand interactions to activate NK cells, but it is also possible that it promotes chemotaxis of the infected cell to uninfected cells that are secreting the XCL1 and may in turn become infected. Indeed, chemotaxis of infected primary human leukocytes toward the chemokines CCL11 (C-C motif chemokine) and CCL19, which also bind to U51A, has been shown to occur in vitro [94]. These experiments also add to the credence of the notion that the binding of certain ligands to U51A results in temporary protection for the virus by demonstrating that U51A was internalized upon binding to the target ligands. Moreover, under normal circumstances, XCL1 triggers apoptosis in CD4+ T cells when it binds to XCR1, which could be detrimental to the longevity of an HHV-6 infection. Taken together, these responses underline how important it is for HHV-6 to counter the initial immune response. 

The viral ligand U83A is produced early in infection and binds CCR5, which blocks binding by CCL5 [94,95]. At the same time, U51A reduces CCL5 expression, resulting in a peak at 24 h post-infection [94] and a subsequently drop. Later in infection, at 6 days and onward, CCL5 is upregulated [58,96]. Early downregulated CCL5 expression might contribute to lower NK cell activation and interruption of the migration of NK cells to HHV-6 infected cells. On the whole, the data indicates that early viral genes seem to lower NK cell activation and allow the virus to spread undetected, but persistent infections may result in activation of NK cells with an altered NK cell response taking the form of atypical chemokine receptor–ligand profiles. In fact, HHV-6B infection decreases the expression of the cellular ligands of several NK receptors (MICB, ULBP1 (UL16 binding protein), ULBP3 and B7-H6) [97]. 

In combination with its expression of U51A and U83A, HHV-6 suppresses the expression of surface proteins that alert immune cells by triggering two major activating receptors on NK cells: NKG2D and NKp30 [98]. Consequently, the ability of NK cells to counteract HHV-6 reactivation and recruit adaptive immune cells is hindered. Experiments point to the presence of at least two early viral proteins that cause the downregulation of the NKG2D ligands through proteosomal degradation at a post-translational level, as well as a separate mechanism that affects the transcription of B7-H6 mRNA, the cellular ligand of the activating receptor NKp30. These findings are corroborated by the drastically reduced clearing efficiency exhibited by NK cells when they were added to cultures of HHV-6 infected cells after 24 h [99]. Moreover, the results suggest that an inefficient NK cell response may have a significantly detrimental impact on the course of infection and the establishment of a persistent infection.

Notably, both HHV-6A and HHV-6B, although with species-specific differences, induced significant modifications in miRNA expression [6]. miRNAs, small non-coding RNA molecules, are known to play an essential role in fine-tuning host immune homeostasis and responses, as miRNA-mediated regulation of gene expression has a profound impact on immune cell development, function, and response to invading pathogens. HHV-6 infection modifies the expression of specific miRNAs involved in development, maturation and effector functions (i.e., miR-146, miR-155, miR-181, miR-223), and on at least 13 miRNAs with recognized role in inflammation and autoimmunity. Also the expression of transcription factors is significantly modified by HHV-6A/6B infection, with an early increase of ATF3 (Cyclic AMP-dependent transcription factor), JUN and FOXA2 (Forkhead Box A2) by both species, whereas HHV-6A specifically induces a 15-fold decrease of POU2AF1 (POU Class 2 Associating Factor 1), and HHV-6B an increase of FOXO1 (Forkhead box protein O1) and a decrease of ESR1 (Estrogen Receptor 1). Interestingly, ATF3 (Activating Transcription Factor 3), upregulated by both viruses, was reported to regulate negatively NK cell functions in MCMV infected mice, by modulating IFNgamma expression [100]. On the other hand, although not yet studied in NK cells, several evidences currently associate PPAR-gamma (peroxisome proliferator-activated receptor gamma) to Th lymphocyte differentiation, B lymphocyte effector functions and cytokine expression [101]. POU2AF1, down-modulated by HHV-6A, was recently reported to induce upregulation of host defense genes, including *IL-6*, in airway epithelium [102]. FOXO1 and ESR1, respectively increased and decreased by HHV-6B, are important regulators of the immune response, being FOXO1 a negative regulator of NK cell maturation and functions [103], whereas ESR1 has been associated to regulation of inflammatory pathways of innate immune cells [104]. JUN is involved in several biological processes as regulators of cell cycle progression, hematopoietic cell differentiation and apoptosis [105,106,107,108], and might be studied in detail in the NK cell context.

## 7. HHV-6 Infection and NK Cell “Memory”

In recent years, NK cell memory, defined as a quantitatively and qualitatively enhanced immune response upon re-challenge, has gained momentum as a topic of interest. For NK cells, two main types of memory exist: (i) In a fashion similar to T cells and B cells, NK cells can exert immunological memory after encounters with stimuli such as haptens or viruses, resulting in the generation of antigen-specific memory NK cells; (ii) NK cells can “remember” an inflammatory cytokine environment that imprints long-lasting non-antigen-specific NK cell effector function [109].

Using an experimental system in which Ly49Hpos NK cells are adoptively transferred into mice lacking this receptor, robust antigen-driven expansion of these Ly49Hpos cells was observed following murine CMV (MCMV) infection [109,110], and after control of the infection, expanded effector NK cells underwent a contraction phase to establish a long-lived and self-renewing “memory” or “adaptive” pool of antigen-specific cells that could be recovered many months following infection in a variety of peripheral tissues. These memory NK cells display a unique transcriptional signature compared to naïve NK cells [110] and functional attributes commonly associated with memory T cells such as secondary expansion, enhanced effector function ex vivo, and increased protection against virus challenge compared to naïve NK cells from uninfected mice [94]. In addition to evidence supporting NK cell memory formation during MCMV infection, several studies suggest that NK cells contribute in secondary immune responses to other viral infections. NK cells previously exposed to herpes simplex virus 2 (HSV-2) or vaccinia virus infection display enhanced IFN-γ production and protection upon re-challenge in a process that is specific to the priming virus, but independent of the adaptive immune system [111,112]. It is tempting to speculate that different types of memory NK cells might be nurtured by different organ environments. 

Distinct patterns of tissue localization have been observed for memory NK cells. Vaccinia virus [113] specific memory NK cells reside in the liver. In influenza VLP (virus like particle)-sensitized mice, memory NK cells isolated from the lungs provided protection against influenza challenge after adoptive transfer [114]. Both splenic and hepatic NK cells from rhesus macaques that were infected with SIV (Simian immunodeficiency virus)-mediated antigen-specific memory responses towards SIV Gag or Env proteins [115]. By contrast, MCMV-specific NK cells are distributed systemically in all organs [110]. Together, these results demonstrate that NK cells, like CD8+ T cells, undergo activation, expansion and contraction in an antigen-specific manner to generate long-lived memory cells in response to viral infection, with a possible tissue specificity. Whether HHV-6-antigen-specific NK cells undergo a similar activation will be an interesting topic for future research. In particular, exploring memory NK responses in the presence of long-term, chronic HHV-6A/B infections could shed light on the disease associations of the viruses.

## 8. Conclusions

NK cell activity is crucial in mounting an early defense against viral infections and in controlling viral reactivation. However, limited data is available on the effects of HHV-6A and HHV-6B on NK cell activity. While it is obvious that functional NK cell deficiencies allow for the development of robust herpesvirus infections, mainly in individuals with genetic predispositions and immunosuppression, it has also been demonstrated that NK cells with a high activation status are involved in incomplete clearance of HHV-6 in the thyroid, brain and uterus [21,46]. The mechanisms behind this dysfunction and high cytotoxicity might be related to the disruptive effects that HHV-6A and -6B are able to induce directly on NK cells, through epigenetic modulation of the host cell, and indirectly via changes in signaling molecules, perhaps as a result of prolonged semi-latent HHV-6 infection. Several cytokines/chemokines are upregulated by HHV-6 reactivation and may contribute to a range of clinical manifestations, including those seen in transplant recipients [35]. In addition, in vitro studies have demonstrated that HHV-6 can modulate host immunity by several mechanisms, including killing lymphocytes and controlling cytokine/chemokine synthesis [94]. Likewise, viral antigens appear to comprise an important barrier to NK-cell-mediated destruction. Abnormal activation of NK cells might also result from the expression of these proteins, but this aspect of HHV-6/NK cell interaction is poorly understood. Together, the cytokines and chemokines induced by HHV-6 infection, as well as viral proteins themselves, may play important roles in both the activation and suppression of the NK cell response, and consequently, in the course of an HHV-6 infection. 

Genetic variations may also influence the development of HHV-6 chronic infections and HHV-6-associated autoimmunity, and may predispose certain individuals to these conditions in part through problems in NK cell functionality [115,116,117,118,119]. In light of this, genetic analysis of patients with chronic HHV-6 infections, such as those reported in cases of HT and idiopathic infertility, could prove useful and may ultimately point to partial underlying NK cell deficiencies [21,46].

NK cells aided by dendritic cells may be the most relevant components of the innate reaction to HHV-6 infection. In addition to their non-specific cytotoxic activity, it is evident that NK cells can take on a novel helper role, in that NK cells may well be exploited for enhancing and rescuing the T-cell response in situations in which the CD4 helper response is affected. In this way, NK cells control the quantity and quality of HHV-6-specific CD8+cytotoxic T lymphocytes, thereby affecting a person’s susceptibility to HHV-6-associated diseases.

Further research is needed to characterize the types of NK cells that are affected by HHV-6 and the range of effects that HHV-6 exerts on NK cells. Future studies may reveal whether suppression of NK cell activity, a particular NK cell receptor repertoire, and/or a disrupted Th1/Th2 ratio is a result of inherent NK cell dysfunction that allows HHV-6 to establish a persistent infection, the NK cell dysfunction is a manifestation of the infection itself, or some combination of these scenarios exists. Consequently, exploration of this topic will help to clarify what kind of role HHV-6 plays in such pathological conditions as HT, female infertility, and even MS, in which NK cell activity is abnormal. Not only will advances in this field shed light on the mechanisms involved in viral persistence and chronic inflammatory states, but it will also open the door for the development of new immunotherapies to treat patients with HHV-6A- and -6B-associated diseases, many of whom would greatly benefit from advances in treatment options. This is particularly relevant in the setting of chronic HHV-6 infections, for which diagnosis and treatment occurs on a limited scale. Since recent studies support a model in which NK cells display virus-specific expansion to form long-lived memory cells that exhibit specific functional recall responses, a better understanding of the role of NK cells in HHV-6 infection could also be useful in determining whether the NK cell compartment can be harnessed in immunization strategies against HHV-6 where no vaccine or specific cure currently exists. 

## Figures and Tables

**Table 1:** HHV-6A and HHV-6B host-interaction characteristics.

Characteristics T	HHV-6A	References	HHV-6B	References
In vitro cell tropism	T lymphocytes, Monocytes Thryocytes Cardiac endothelial cells Lymphatic endothelial cells Mesothelial cells Glial cells Fibroblasts Natural killer cells	Caruso 2003 [7] Caruso 2009 [8] Caselli 2015 [9] Gu 2011 [10] Li 2012 [11] Rizzo 2017 [6]	T lymphocytes Monocytes Lymphatic endothelial cells Glial cells Fibroblasts-limited Natural killer cells	Caruso 2009 [8] Harberts 2011 [12] Robert 1996 [13]
In vivo cell tropism *	Endometrial epithelium Tonsillar crypts epithelium Mucous, serous, and ductal cells of salivary glands Liver bile duct epithelium Large bowel crypt epithelium	Marci 2016 [21] Roush 2001 [15] Fox 1990 [16] Pilmore 2009 [22]	Adenomatous polyps Renal tubular epithelial cells Hepatocytes Portal vein endothelium, Tonsillar crypts epithelium Pneumocytes Thyocytes Cardiac endothelium	Halme 2013 [23] Helantera 2008 [24] Ozaki 2001 [25] Kuribayashi 2006 [26] Aita 2001 [27] Roush 2001 [21] Pitalia 1993 [28] Sultanova 2017 [29] Kuhl 2005 [30]
Primary infection	Asymptomatic (Largely unknown)		Exhantem subitum Febrile seizure	De Bolle 2005 [2]
Congenital infection	Unknown consequence		Unknown consequence	
Immuno-compromised host	Unknown		Encephalitis Graft versus host disease Delayed platelet engraftment	Clarck 2003 [31]
Host-genome integration	Yes		Yes	
Host cell receptor	CD46	Santoro 2005 [4]	CD134	Tang 2013 [5]

* In addition to those listed in vitro. HHV = Human herpesvirus.

**Table 2:** HHV-6 proteins.

HHV-6 Component	HHV-6 Proteins
Membrane	Glycoprotein H (U48)
	Glycoprotein B (U39)
	Glycoprotein Q (U100)
	Myristylated virion protein (U71)
Tegument	Antigenic virion protein (U11)
	Phosphoprotein pp85 (U14)
	Virion transactivator (U54)
Capsid protein	Major capsid protein (U57)
Non-structural proteins	Polymerase processivity factor (U27)
	Parvovirus rep homolog (U94)
	DNA polymerase (U38)
	Immediate early protein 1 (U90)
	Tail anchored membrane protein (U24)

**Table 3:** HHV-6A and HHV-6B proteins involved in NK cell activation control.

**HHV-6A**			
**Viral Protein**	**Host Protein**	**Effect**	**References**
U51A	XCL1	Block CCR7 binding Decrease NK cell activation Increase chemotaxis of infected cells to uninfected cells	Catusse 2008 [94]
U83A	CCR5	Block CCR5 binding Decrease NK cell activation	Catusse 2008 [94] Caruso 2003 [4] Milne 2000 [96]
**HHV-6B**			
**Viral Protein**	**Host Protein**	**Effect**	**References**
E protein	Decrease MICB, ULBP1, ULBP3 expression	Block NKG2D activation	Schmiedel 2016 [98]
E protein	Decrease B7-H6 expression	Block Nkp30 activation	Schmiedel 2016 [98]
